# Development trends and knowledge framework of artificial intelligence (AI) applications in oncology by years: a bibliometric analysis from 1992 to 2022

**DOI:** 10.1007/s12672-024-01415-0

**Published:** 2024-10-16

**Authors:** Murat Koçak, Zafer Akçalı

**Affiliations:** Department of Medical Informatics, Faculty of Medicine, Baskent University, Taşkent Caddesi (Eski 1. Cadde) 77. Sokak (Eski 16. Sokak) No:11, 06490 Bahçelievler, Ankara Turkey

**Keywords:** Oncology, Cancer, Artificial intelligence, Deep learning, Neural network

## Abstract

**Purpose:**

Oncology is the primary field in medicine with a high rate of artificial intelligence (AI) use. Thus, this study aimed to investigate the trends of AI in oncology, evaluating the bibliographic characteristics of articles. We evaluated the related research on the knowledge framework of Artificial Intelligence (AI) applications in Oncology through bibliometrics analysis and explored the research hotspots and current status from 1992 to 2022.

**Methods:**

The research employed a scientometric methodology and leveraged scientific visualization tools such as Bibliometrix R Package Software, VOSviewer, and Litmaps for comprehensive data analysis. Scientific AI-related publications in oncology were retrieved from the Web of Science (WoS) and InCites from 1992 to 2022.

**Results:**

A total of 7,815 articles authored by 35,098 authors and published in 1,492 journals were included in the final analysis. The most prolific authors were Esteva A (citaition = 5,821) and Gillies RJ (citaition = 4288). The most active institutions were the Chinese Academy of Science and Harward University. The leading journals were Frontiers ın Oncology and Scientific Reports. The most Frequent Author Keywords are "machine learning", "deep learning," "radiomics", "breast cancer", “melanoma” and "artificial intelligence," which are the research hotspots in this field. A total of 10,866 Authors' keywords were investigated. The average number of citations per document is 23. After 2015, the number of publications proliferated.

**Conclusion:**

The investigation of Artificial Intelligence (AI) applications in the field of Oncology is still in its early phases especially for genomics, proteomics, and clinicomics, with extensive studies focused on biology, diagnosis, treatment, and cancer risk assessment. This bibliometric analysis offered valuable perspectives into AI's role in Oncology research, shedding light on emerging research paths. Notably, a significant portion of these publications originated from developed nations. These findings could prove beneficial for both researchers and policymakers seeking to navigate this field.

## Introduction

Artificial intelligence is based on creating machines that think like humans and is seen as the apotheosis of science [1]. Artificial intelligence (AI), which has emerged in different fields in the twenty-first century, has become a trend in many fields, such as medicine, science, and business [2].

The integration of artificial intelligence (AI) into cancer treatment has also gained momentum, with AI-powered predictive models showing promise in identifying patients who are most likely to respond to specific therapies. Recent studies have demonstrated the potential of AI to predict response to checkpoint inhibitors in non-small cell lung cancer [3], predict response to chemotherapy in breast cancer [4], identify high-risk patients who may benefit from early intervention [5], develop personalized treatment plans for patients with rare cancers such as pancreatic cancer [6], and predict treatment outcomes in patients with melanoma [7]. As the field of cancer treatment continues to evolve, it is essential that we stay up-to-date with the latest advancements and explore the potential applications of AI in predicting response to cancer therapies.

It is possible to categorize medical data that can be assessed with deep learning/machine learning/artificial intelligence into genetic, imaging, and clinical data. The images may be real-size photographs of visible parts of the human body and internal parts of the human body obtained by devices such as computed tomography, x-ray, magnetic resonance, and endoscopic devices. Radiomics is a research field that applies advanced image analysis techniques to extract quantitative features from medical imaging data.. In 2009, “Radiogenomics: Creating a link between Molecular Diagnostics and Diagnostic Imaging” [8] and “Radiomics: Images Are More than Pictures, They Are Data” [9] in, the term radiomics started to appear in the literature.

Competitions using The Pascal Visual Object Classes Challenge and Imagenet datasets have been held worldwide to recognize highly accurate images by electronic systems. In 2012, AlexNet software, which participated in the competition under the name Supervision, won this competition by a large margin, and the paper "ImageNet classification with deep convolutional neural networks," published by the authors [10], was an important milestone. Please note that, Ilya Sutskever is a major contributor to ChatGPT, a large language model based AI software. The article "Radiomics: images are more than pictures, they are data” [9], drew attention to the fact that meaningful results can be obtained primarily in cancer patients by converting radiologic images into numerical data. Publications on the ability of Alexnet-type software to successfully evaluate patient-related images have started to appear since 2017 and have increased steadily.

Magnified images of cells in the human body tissues can be obtained with devices such as endoscopy, or cells extracted from the body by biopsy can be examined by various processes and staining. The field that deals with microscopic images of tissues are called pathomics [11]. There are very few publications using the term pathomics.

Genetic data can be related to DNA gene sequences or protein structures. The field dealing with gene sequences is called genomics and the field dealing with protein structures is called proteomics. The highly cited publications on gene sequencing and cancer started even before radiomics and received many citations [12]. Genetic data can be about DNA gene sequences or protein structures. The field that deals with gene sequences is called genomics, and the field that deals with protein structures is called proteomics. Highly cited publications on gene sequencing and cancer started even before radiomics [12, 13]. Although not cited as genomics, publications on proteomics started appearing before radiomics [14].

While the name clinicomics seems appropriate for evaluating clinical data with machine learning [15–17], the term has only recently been used. In the USA., clinical data obtained from hospitals' electronic health records (EHR) are anonymized/de-identified in a CancerLinq database and re-recorded so that meaningful conclusions can be drawn and used by users who want to benefit from it [18, 19]. Unlike the SEER (Surveillance, Epidemiology, and End Results) database (https://seer.cancer.gov, an extensive anonymized cancer patient database), where fewer parameters about a patient are stored, it should be considered to allow for the evaluation of individualized diagnoses and treatments. Clinicomics may attempt to extract meaningful data from text based clinical records and is expected to utilize large language model AI frameworks.

Since it is necessary to use one of the research methods appropriate for evaluation and prediction, bibliometric analysis is one of the analyses that serves this purpose. Bibliometrics is used as a type of analysis that systematically analyzes datasets [20]. This type of analysis requires a systematic literature review and meaningful structuring of a large dataset [20]. Bibliometric analysis is a method used to analyze the studies conducted in a specific field, and its use has increased in recent years.

While bibliometric analysis in this concept is seen as inevitable for many fields [21], it is necessary to examine academic studies on the impact of artificial intelligence applications in medicine through bibliometric analysis. This is because AI's increasing popularity and widespread use cause the subject to become a need in terms of health. This study analyzed 7923 articles published between 1992 and 2022 with the R program, biblioshiny package program, and VOSviewer software [22]. The article contributed to determining the techniques used in the cumulative information in the relevant research, thematic analysis, and central trends. It sheds light on the research to be conducted. In this context, the study will first include the literature on artificial intelligence and cancer, then the data used and the research method will be mentioned.

Karger [23] provides a bibliometric analysis of research on AI for cancer detection, emphasizing its growth and potential for early detection. Khanam and Kumar [24] explore the recent applications of AI algorithms, including machine learning and deep learning, in the early detection of various cancers. Pacurari et al. [25] focus on using AI techniques, such as support vector machines and neural networks, to detect and classify lung, breast, and brain cancers using medical imaging. Overall, these papers highlight the importance of AI in improving Oncology and emphasize the need for further research in this field.

The objective of this research is to address specific inquiries regarding the utilization of Artificial Intelligence (AI) in Oncology.

- Sources: Which scientific journals are most influential in the field?
- Researhers: Which Authors are the most influential in the field of Artificial Intelligence (AI) applications in Oncology?
- Papers: Which papers about Artificial Intelligence (AI) applications in Oncology?
- Keywords: What are the most popular authors keywords in Artificial Intelligence (AI) applications in Oncology research?
- How have the themes of Artificial Intelligence (AI) applications in Oncology evolved?
- Funding: Which fundings are the most influential in the field of Artificial Intelligence (AI) applications in Oncology?

## Method

The relevant subject is examined statistically and mathematically in Bibliometrics, and a framework is drawn for the course. In addition, bibliometric analysis can also reveal the effectiveness of studies conducted through statistical data [26]. The data for this study were retrieved from the online database Web of Science on 21.11.2023. The reasons for using the Web of Science database instead of Scopus Google Scholar for bibliometric analysis are that it is the most extensive database in abstracts and literature, it can produce information with better actions, decisions, and results [27], it is a valuable resource for bibliometric studies, and it offers a comprehensive perspective on publications in the fields of science, technology, art, medicine, and social sciences. The Web of Science database used in bibliometric analysis is preferred [28].

The search strategy of this study is as follows: While obtaining the data of the study, the first step was to search the Web of Science database (TI = ("deep-Learn*" OR "machine learn*" OR "deep learn*"OR "artificial intelligence" OR "artificial neural network*" OR "deep neural network*" OR radiomics OR pathomics)) AND (AB = (melanoma OR cancer OR malignancy OR leukemia OR lymphoma OR neoplasia OR SEER* OR "Surveillance, Epidemiology, and End Results" OR CancerLinQ*)) AND (PY = 1992–2022) AND (DT = Article).

Data from databases were directly accessed to retrieve metadata from chosen documents, encompassing details like active authors, journal sources, countries, institutions, and funding sources. The analysis involved employing tools such as Bibliometrix[29] package (version 3.1.4, http://www.bibliometrix.org) in R software (version 3.6.3), VOSviewer [22], and Litmaps [30] to generate comprehensive bibliometric insights. Initially, raw data in plain text format was loaded and processed, involving calculations and visual representations of metadata comprising sources, authors, and citations. This was followed by intricate analyses focusing on clustering and the conceptual framework encompassing intelligence and society. Additionally, a comprehensive bibliometric evaluation was carried out, encompassing co-authorship and keyword co-occurrence using the VOSviewer software [22].

## Results

When 7923 articles were reviewed, although the importance of genetic data in cancer diagnosis is significant, there are relatively few publications in machine learning and genomics fields, comprising only 2.2% (174 articles) of the studies in this work. There are only 12 articles related with pathomics, and deep learning in WOS database. The term 'Clinicomics' has not yet been established and has not found a place within the article set of this study. When searching for the term 'Clinical informatics,' only four articles were found; 'Clinical data extraction' yielded 1.6% (124 articles), 'SEER' resulted in 0.90% (71 articles), and 'NLP' led to 28 articles. Only 1 article containing the term 'CancerLing' [31] was found. Only three articles can be found when the keywords 'CancerLinq' and 'machine learning' are searched across all fields in the WOS database. As 1716 articles were found with the term 'Radiomics' and 2215 articles with the term 'image,' we can conclude that the most commonly used datasets related to the topic of this article are primarily in the form of images.

### General information

The findings of the bibliometric analysis conducted within the scope of the study will be presented in this section.

Table 1 provides fundamental details about the dataset, encompassing its size, descriptive statistics, content overview, and statistics regarding authorship and collaborative efforts. It has 7923 documents over the timespan of 1992–2022. 7923 journal articles were extracted from the Web of Science database. This downloaded database was then thoroughly analyzed and examined with the help of the Bibliometrix [29] and R software (2023) application. At the early stage of this study, a descriptive analysis was provided to examine the details of the work published in this area (Table 1).

**Table 1:** General information about the publications analyzed in the Artificial Intelligence (AI) and Oncology Dataset

Description	Results
Main Information About Data	
Timespan	1992:2022
Sources (Journals, Books, etc.)	1592
Documents	7923
Annual Growth Rate %	19.34
Average citations per doc	24
Document Contents	
Keywords Plus (ID)	7443
Author's Keywords (DE)	10,966
Authors	
Authors	35,198
Authors of single-authored docs	120
Co-Authors per Doc	7.95
International co-authorships %	29.48

Bibliometrix & R software (2023)

**Fig. 1:**
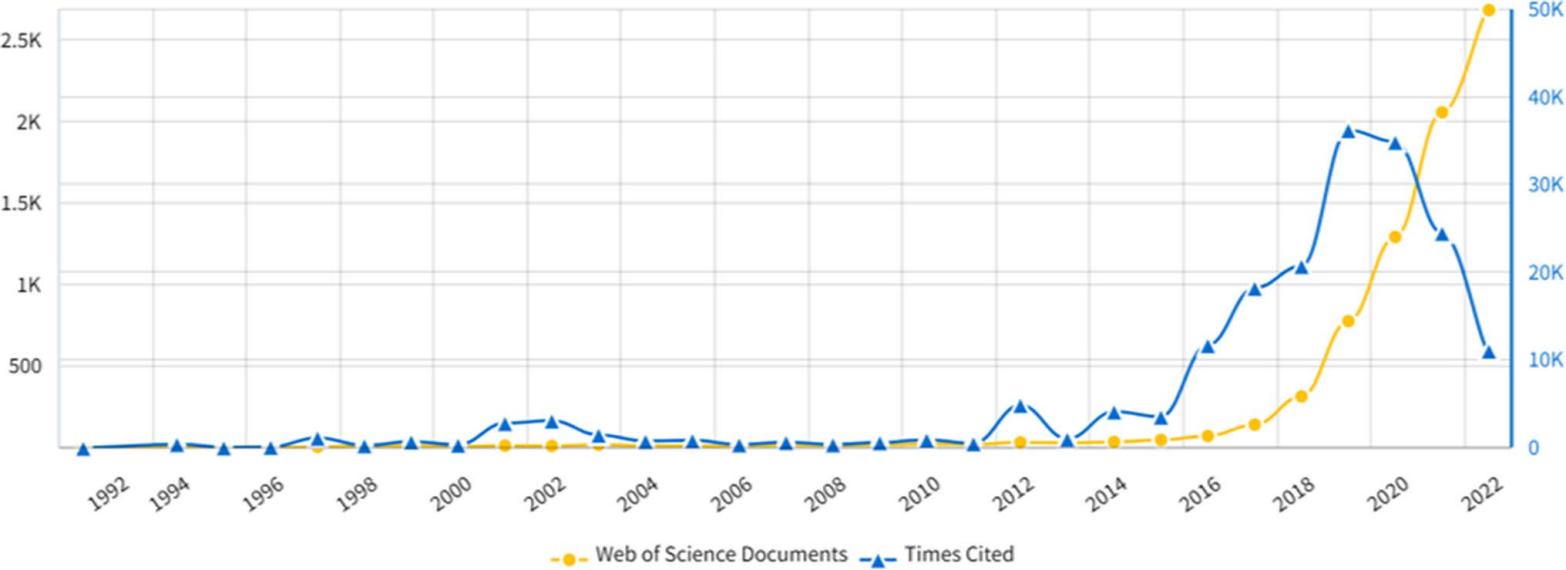
Distribution of WOS articles and Citation on Artificial Intelligence (AI) and Oncology between 1992 and 2022 (n = 7923) (InCites, 2023)

Table 1 shows that the analyzed studies consist of 7923 publications published between 1992 and 2022 and are based on the statistics of publications on oncology using artificial intelligence methods. Figure 1 shows the quantity of AI-related articles within the oncology domain. Overall, there's been a consistent upward trend in publications from 1992 to 2022. Particularly in the last decade, there has been a surge in global interest within the research field. Between 1994 and 2014, publications remained relatively low and steady. However, since AI gained prominence around 2016, there has been a notable and substantial increase in the number of publications.

**Fig. 2:**
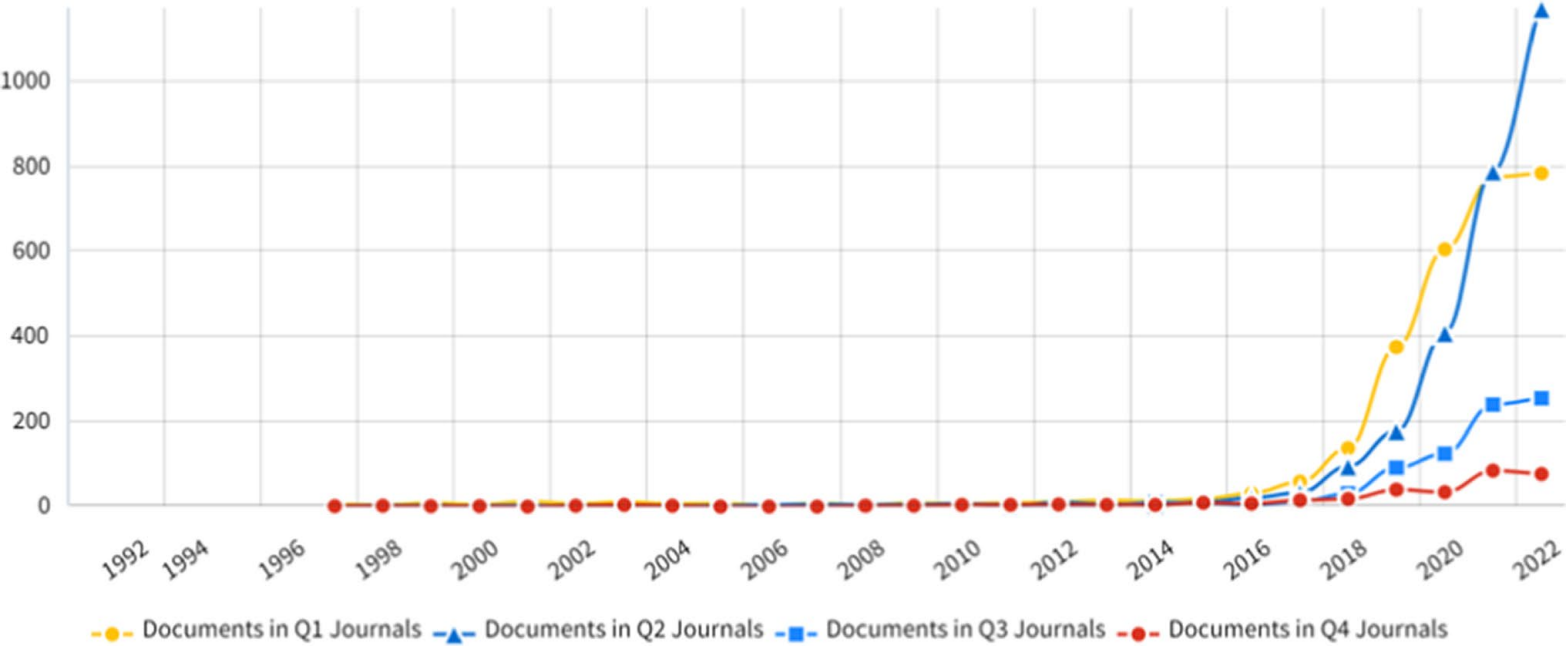
Distribution of documents in Q1, Q2, Q3, and Q4 Journals on Artificial Intelligence (AI) and Oncology between 1992 and 2022 (InCites, 2023)

The trend of the journal impact factor quartile of articles is shown in Fig. 2. From 2016 to 2021, the number of documents in Q1 articles showed a fast growth trend; after 2021, the number of documents in Q1 has gone steadily. For the first time in 2021, the number of articles in the Q2 category exceeded that in the Q1 category. This means that higher-quality journals are starting to lower acceptance rates in this field. The lowest rate in this area is for articles in the Q4 category. This indicator shows the popularity of this field.

The Web of Science was used to meticulously define and classify citation topics. All findings from the search query were included in the review without further filtration. The citation topics were narrowly focused and categorized according to the recently published classifications by the Web of Science, encompassing over 2500 detailed citation topics. This classification operates hierarchically below the Web of Science subject categories and citation topics at a broader level, enabling a precise and unbiased evaluation of the technologies utilized in the search query.

**Fig. 3:**
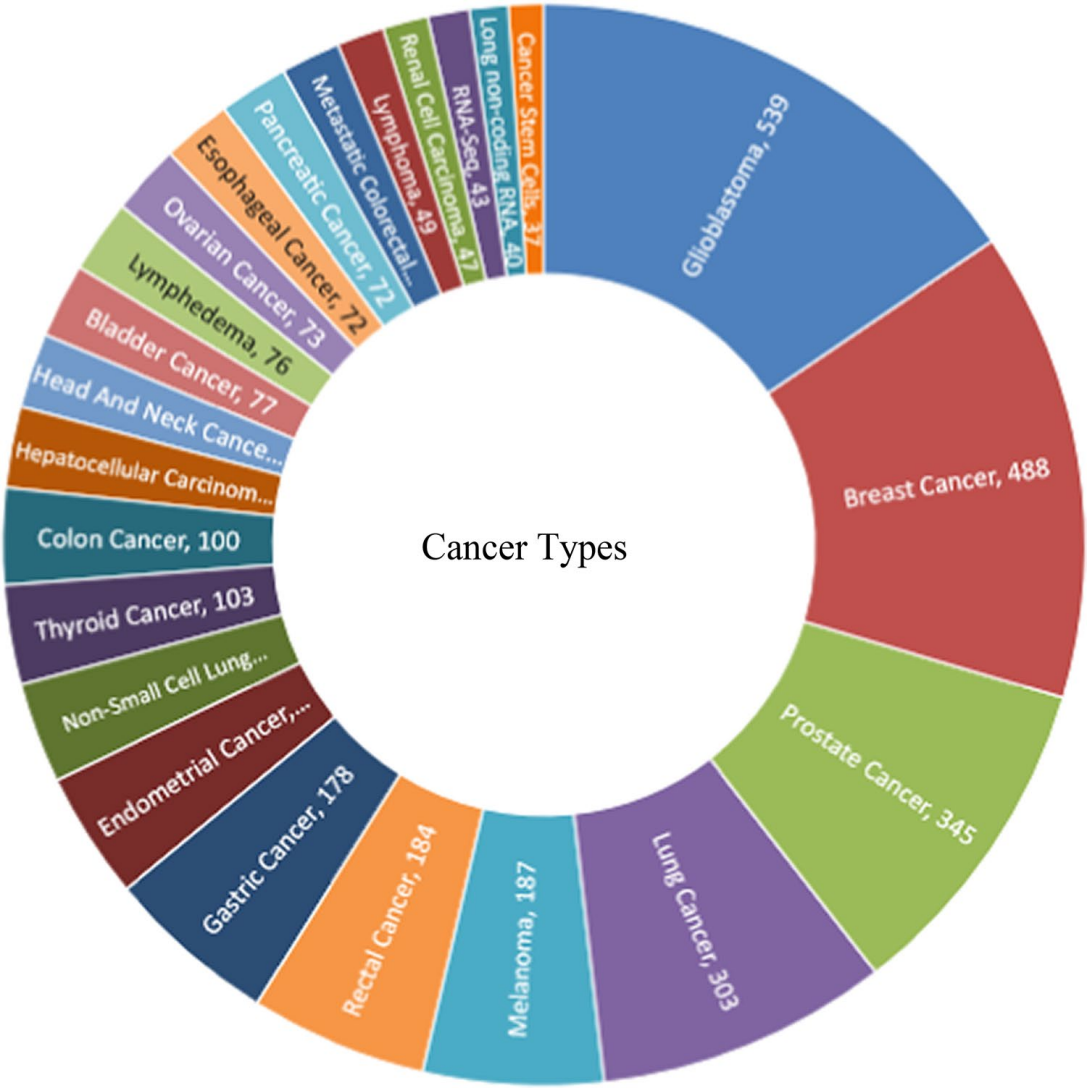
The most common types of diseases mentioned in the articles were Artificial Intelligence and Oncology (Web of Science, 2023)

Our datasets were determined and ranked based on the citation topic micro criteria in the WoS Citation index [27]. The most represented citation topics micro based on our datasets were "glioblastoma", "breast cancer," "prostate cancer," "lung cancer," “melanoma” "rectal cancer," and "gastric cancer." Various cancer types are distributed among the article publications related to cancer research. The analysis of cancer types investigated provides insight into the present landscape of cancer research employing artificial intelligence techniques. As depicted in Fig. 3, within this decade, the most prevalent studies have focused on glioblastoma (539 articles) and breast cancer (488 articles), with prostate cancer (345 articles) following closely in frequency.

### Authors

**Fig. 4:**
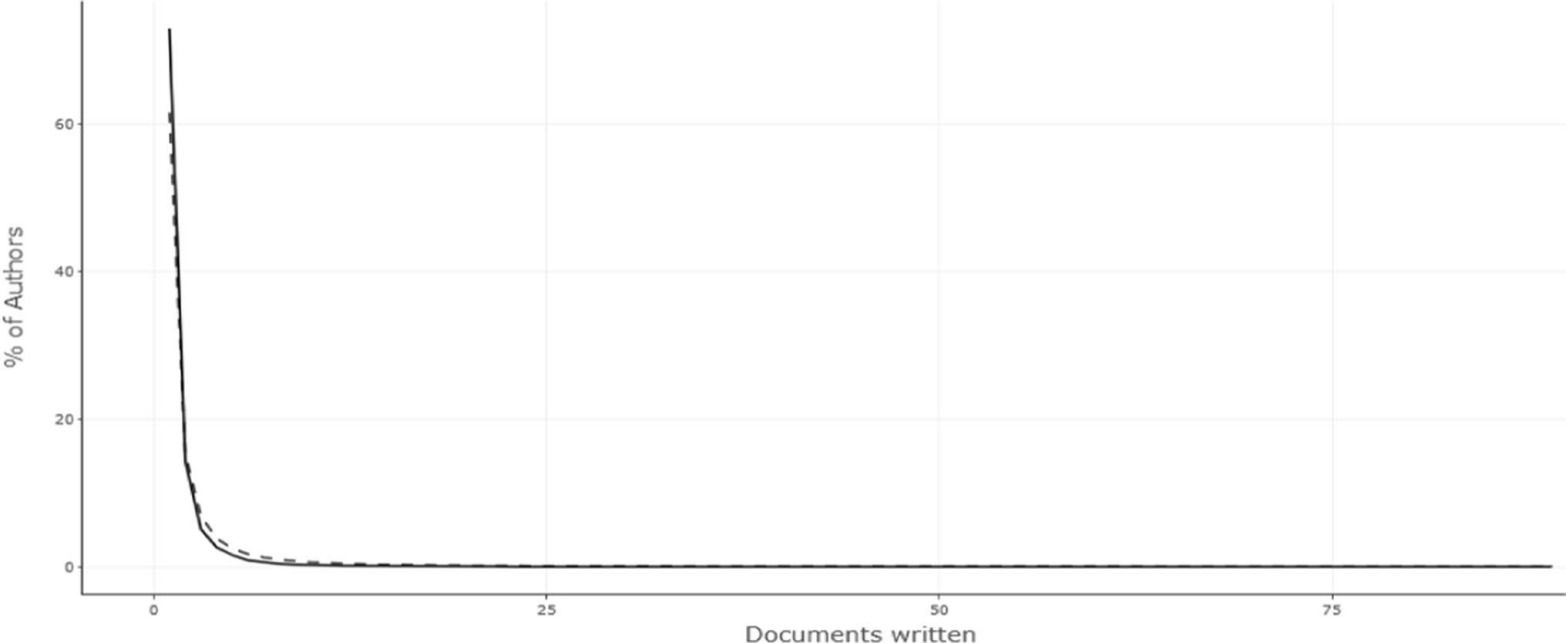
Lotka's Law and Author Productivity Rate (Bibliometrix & R software, 2023)

In Table 2 and Fig. 4, Lotka's Law reveals the quantitative distribution of the publications of authors who contribute to the literature on a particular subject in the literature of that field. With this, the scientific productivity of the authors was tried to be revealed. Lotka's Law predicts that 70% of the authors who publish on a subject contribute to the subject with one publication, 15% with two publications, and 7% with three publications [32]. It can be seen that the data obtained as a result of the analysis also complies with Lotka's Law. According to Table 2, 78% of the authors made one publication, 13% made two, and 4% made three.

**Table 2:** Lotka's Law and Author Productivity Ratio

Documents written	No. of authors	Proportion of Authors
1	25,587	0.729
2	4999	0.142
3	1799	0.051
4	921	0.026
5	553	0.016
6	313	0.009
7	222	0.006
8	136	0.004
9	102	0.003
10	99	0.003

Bibliometrix & R software (2023)

**Fig. 5:**
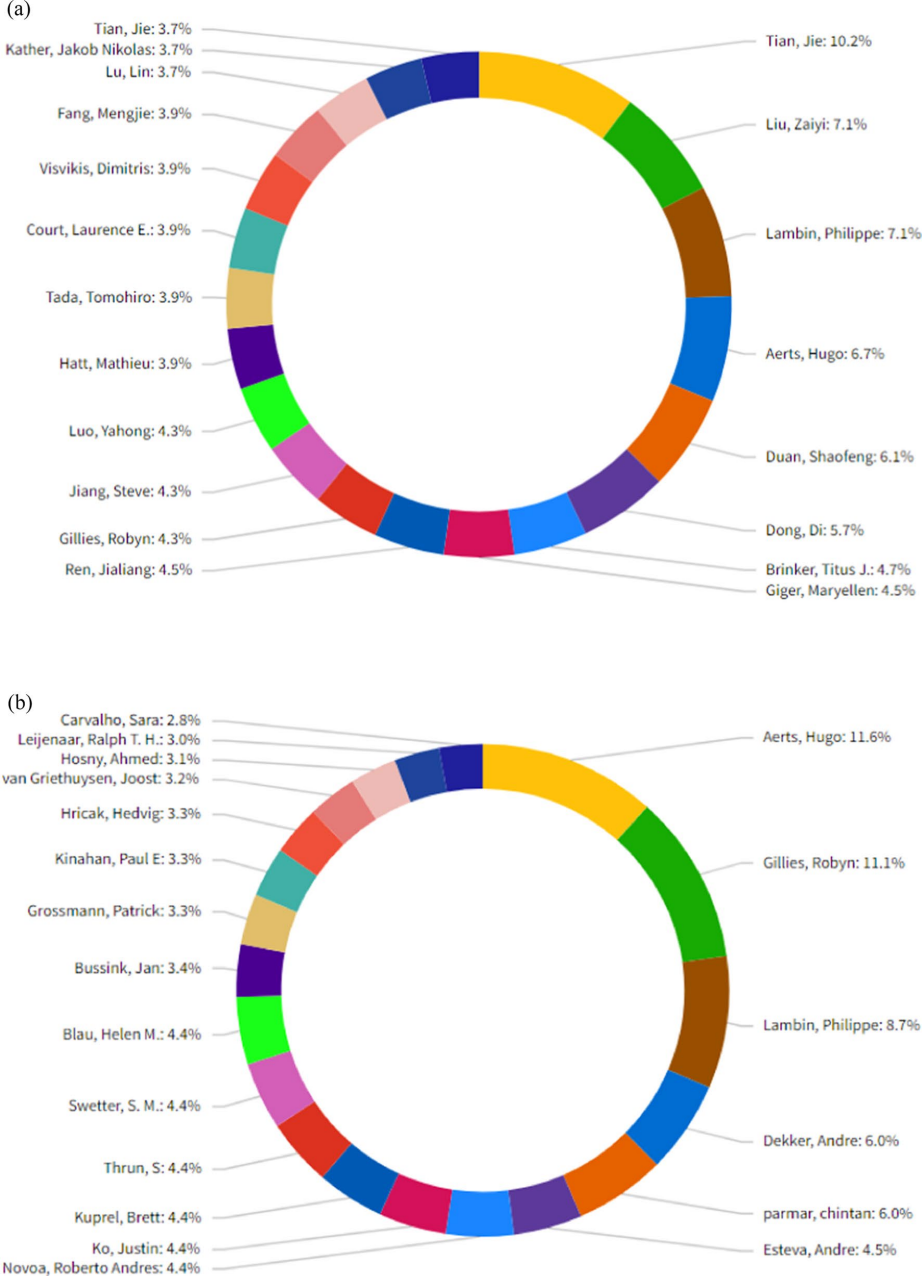
**a** Top 20 Authors published the most AI articles in oncology from 1992 to 2022. **b** Top 20 Authors published the most AI citations in oncology from 1992 to 2022 (InCites, 2023)

Figure 5a shows the main statistical characteristics of the Top 20 authors ranked by number of articles. When the graph is analyzed, Tian, Jie, and Liu Zaiyi are the leading authors working on artificial intelligence and Oncology. The author's publications cover 17.5 percent of the total publications.

Figure 5b shows the main statistical characteristics of the Top 20 authors ranked by number of citations. When the graph is analyzed, Aerts Hugo and Gillies Robyn are the leading authors working on artificial intelligence and oncology. The author's number of citations covers 22.7 percent of the total citations.

**Fig. 6:**
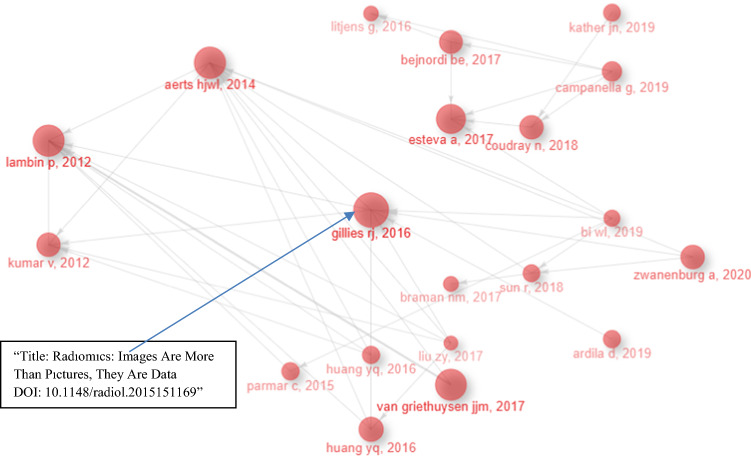
Historiography of AI articles in oncology from 1992 to 2022 (Bibliometrix & R software, 2023)

Figure 6 shows the bibliometric historiography using the science mapping tool bibliometrix [33]. Initially, this mapping process establishes the historical direct citation network starting from the most-cited work, subsequently visualizing the network in chronological order [34]. The subsequent subsections will detail these networks, proceeding from the earliest to the most recent. As seen in Fig. 6, the radomics article by Gillies [9], Robyn is one of the first articles to show the importance of using artificial intelligence in cancer detection. Therefore, it is at the center of the Historiography graph.

### Papers

The top 25 most widely cited articles are presented in Table 3. The article, Dermatologist-level classification of skin cancer with deep neural networks, was published by Esteva A in Nature in 2017 (Total citations = 5900), followed by Gillies [9] in Radiology in 2016 (total citations = 4362), and Lambin [35] published in the European Journal of Cancer in 2012 with 3018 citations. Hence, an annual average citation has been included in Table 3 to aid researchers in promptly identifying recently published highly cited papers. These high-impact publications, in essence, offer a swift overview of the field and expand researchers' perspectives.

**Table 3:** Distribution of Most Cited AI Articles in Oncology from 1992 to 2022

Paper	Article Title	No. of citation	DOI	Journal	WoS categories
Esteva et al., 2017	Dermatologist-level classification of skin cancer with deep neural networks	5900	10.1038/nature21056	Nature	Multidisciplinary Sciences
Gillies et al., 2016	Radiomics: Images Are More than Pictures, They Are Data	4362	10.1148/radiol.2015151169	Radiology	Radiology
Lambin et al., 2012	Radiomics: Extracting more information from medical images using advanced feature analysis	3018	10.1016/j.ejca.2011.11.036	Eur. J. Cancer	Oncology
Aerts et al., 2014	Decoding tumour phenotype by noninvasive imaging using a quantitative radiomics approach	2958	10.1038/ncomms5006	Nat. Commun	Multidisciplinary Sciences
Van Griethuysen et al., 2017	Computational Radiomics System to Decode the Radiographic Phenotype	2725	10.1158/0008-5472.CAN-17-0339	Cancer Res	Oncology
Khan et al., 2001	Classification and diagnostic prediction of cancers using gene expression profiling and artificial neural networks	1897	10.1038/89044	Nat. Med	Biochemistry & Molecular Biology
Shipp et al., 2002	Diffuse large B-cell lymphoma outcome prediction by gene-expression profiling and supervised machine learning	1796	10.1038/nm0102-68	Nat. Med	Biochemistry & Molecular Biology
Bejnordi et al., 2017	Diagnostic Assessment of Deep Learning Algorithms for Detection of Lymph Node Metastases in Women With Breast Cancer	1466	10.1001/jama.2017.14585	JAMA-J. Am. Med. Assoc	Medicine, General & Internal
Kumar et al.,2012	Radiomics: the process and the challenges	1374	10.1016/j.mri.2012.06.010	Magn. Reson. Imaging	Radiology
Zwanenburg et al., 2020	The Image Biomarker Standardization Initiative: Standardized Quantitative Radiomics for High-Throughput Image-based Phenotyping	1367	10.1148/radiol.2020191145	Radiology	Radiology
Coudray et al., 2018	Classification and mutation prediction from non-small cell lung cancer histopathology images using deep learning	1243	10.1038/s41591-018-0177-5	Nat. Med	Biochemistry & Molecular Biology
Huang et al., 2016	Development and Validation of a Radiomics Nomogram for Preoperative Prediction of Lymph Node Metastasis in Colorectal Cancer	1151	10.1200/JCO.2015.65.9128	J. Clin. Oncol	Oncology
Malta et al., 2018	Machine Learning Identifies Stemness Features Associated with Oncogenic Dedifferentiation	1009	10.1016/j.cell.2018.03.034	Cell	Biochemistry & Molecular Biology
Campanella et al., 2019	Clinical-grade computational pathology using weakly supervised deep learning on whole slide images	899	10.1038/s41591-019-0508-1	Nat. Med	Biochemistry & Molecular Biology
Johnson et al., 2019	Survey on deep learning with class imbalance	892	10.1186/s40537-019-0192-5	J. Big Data	Computer Science
Ardila et al., 2019	End-to-end lung cancer screening with three-dimensional deep learning on low-dose chest computed tomography	828	10.1038/s41591-019-0447-x	Nat. Med	Biochemistry & Molecular Biology
Sirinukunwattana et al., 2016	Locality Sensitive Deep Learning for Detection and Classification of Nuclei in Routine Colon Cancer Histology Images	706	10.1109/TMI.2016.2525803	IEEE Trans. Med. Imaging	Engineering, Biomedical
Ye et al., 2013	Predicting hepatitis B virus-positive metastatic hepatocellular carcinomas using gene expression profiling and supervised machine learning	699	10.1038/nm843	Nat. Med	Biochemistry & Molecular Biology
Bi et al., 2019	Artificial intelligence in cancer imaging: Clinical challenges and applications	698	10.3322/caac.21552	CA-Cancer J. Clin	Oncology
Sun et al., 2018	A radiomics approach to assess tumour-infiltrating CD8 cells and response to anti-PD-1 or anti-PD-L1 immunotherapy: an imaging biomarker, retrospective multicohort study	638	10.1016/S1470-2045(18)30,413-3	Lancet Oncol	Oncology
Parmar et al., 2015	Machine Learning methods for Quantitative Radiomic Biomarkers	610	10.1038/srep13087	Sci Rep	Multidisciplinary Sciences
Yao & Liu, 1997	A new evolutionary system for evolving artificial neural networks	591	10.1109/72.572107	IEEE Trans. Neural Netw	Artificial Intelligence
Litjens et al., 216	Deep learning as a tool for increased accuracy and efficiency of histopathological diagnosis	588	10.1038/srep26286	Sci Rep	Multidisciplinary Sciences
Rajpurka et al., 2018	Deep learning for chest radiograph diagnosis: A retrospective comparison of the CheXNeXt algorithm to practicing radiologists	545	10.1371/journal.pmed.1002686	PLos Med	Medicine, General & Internal
Bera et al., 2019	Artificial intelligence in digital pathology—new tools for diagnosis and precision oncology	525	10.1038/s41571-019-0252-y	Nat. Rev. Clin. Oncol	Oncology

Bibliometrix and R software (2023)

For this study, Litmaps, an advanced science discovery platform known for its visual citation navigation, has been utilized. This platform offers an interface facilitating the exploration of scientific literature, enabling researchers to delve into the research terrain and uncover articles intricately linked within maps. Litmaps also presents convenient options for swiftly importing articles through various means such as reference manager, keyword search, ORCID ID, DOI, or by utilizing a seed article [36].

**Fig. 7:**
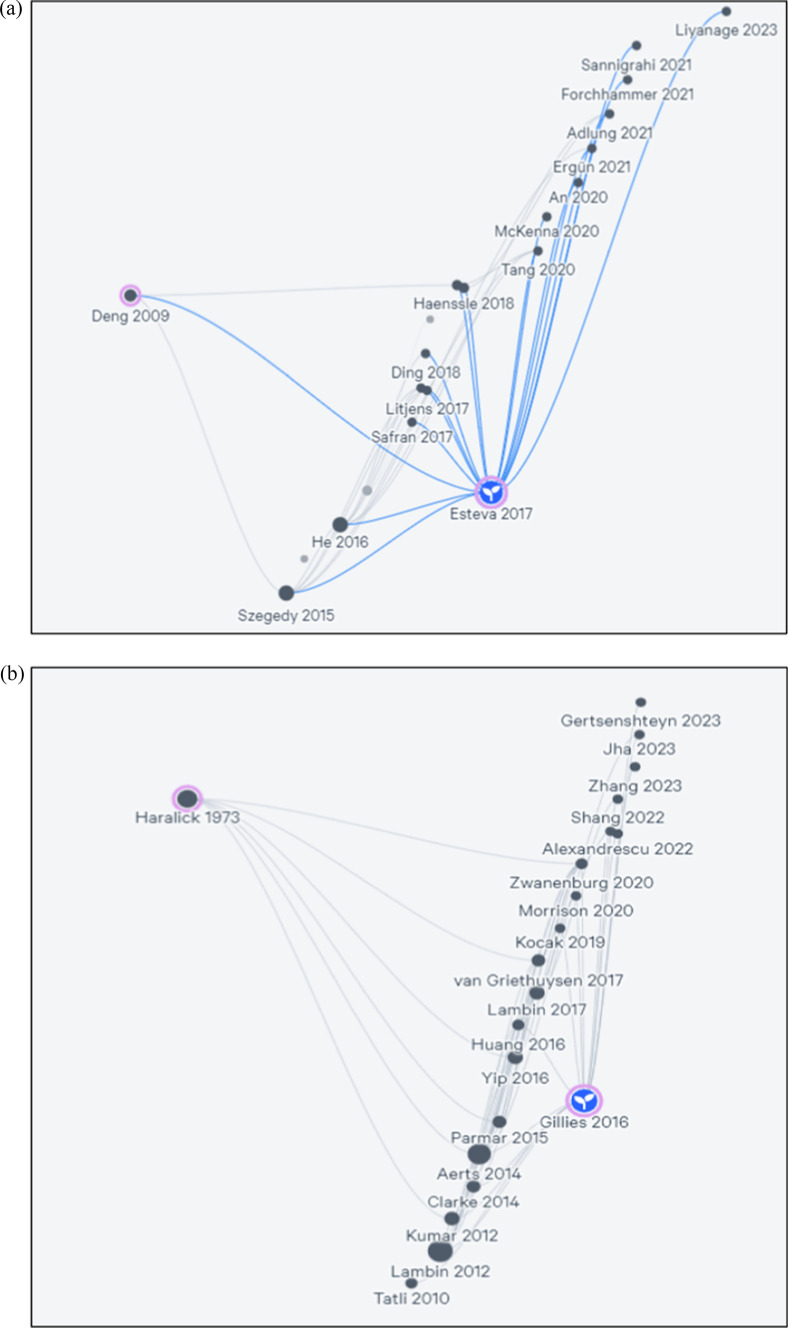
**a** Seed Maps of Esteva [37]. **b** Seed Maps of Gillies [9] (Litmaps, 2023)

Litmaps helps researchers do the literature review very briefly and systematically. It helps find related or relevant studies through the seed paper. This will include some of your Seed Article's direct references and citations and some of their citations and references. Litmaps provides functionalities for visualizing literature maps that encompass pivotal articles relevant to specific research fields through diverse visualization modes. Notably, papers with higher citation counts are represented with larger circles, where the size of the node correlates proportionally to the logarithm of the citation count. In Fig. 7a, b, Seed Maps show the top 20 citations and references related to a single article.

### Sources (Journals)

In Fig. 7, Bradford's Law divides journals into three main classes and helps to find the core journals. First formulated in 1934, Bradford's Scatter Law "describes the scatter or distribution of literature on a particular topic across journals" [34]. According to this law, there should be an inverse relationship between the number of studies published on a topic and the number of journals in which they are published. Journals are divided into regions by ranking them according to the number of studies they publish. Although the number of journals in each region is not equal, the total number of publications in the regions will be equal. Because the productivity of journals is different from each other [38]. As a result of the analysis, it was determined that the journals were divided into three regions. Figure 7 shows the sources in the first region.

**Fig. 8:**
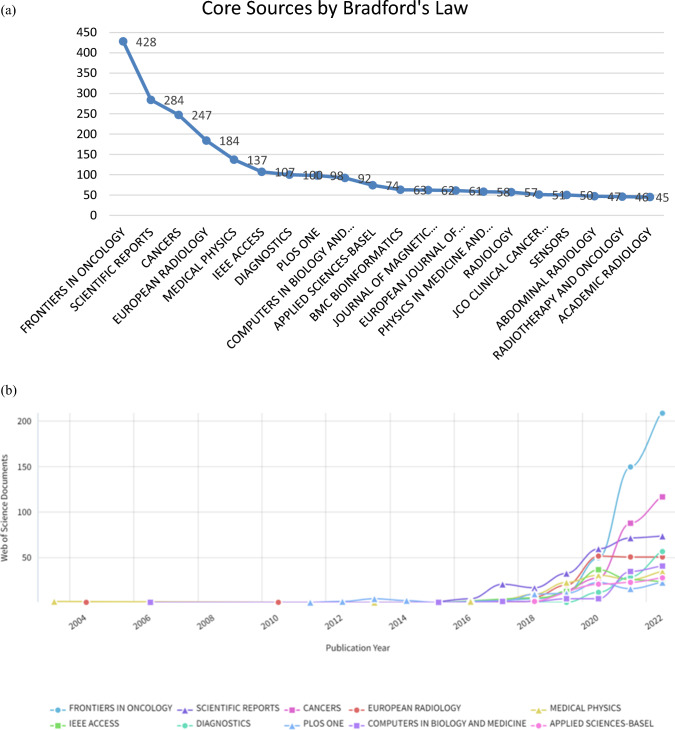
**a** Distribution of the scientific journals publishing the most articles in artificial intelligence and Oncology according to Bradford's law. **b** Distribution of the journals with the most articles by year (Bibliometrix & R software, 2023)

Figure 8a, b shows the trend of AI articles in oncology journals and reveal their decreasing or increasing trend over time in oncology journals. The results in Fig. 8a, b shows the distribution of the journals with the most articles by year. Three leading journals publish the most articles on artificial intelligence and Oncology: Frontiers in Oncology (428 articles) and Scientific Reports and Cancer (284 and 247 articles, respectively). Although these three prominent journals contributed to 12% of the total articles, the remaining publications are widely distributed. This suggests that apart from the top three journals that publish the majority of insights in this research domain, a diverse array of other sources also significantly contributes to the literature.

When we look at the change in scientific journals over the years, it was first published in Scientific Reports in 2015. After 2020, a significant increase in publications in Artificial Intelligence (AI) applications in Oncology was observed. Frontiers in Oncology journal is leading this increase.

As shown in Fig. 8b, Scientific Journals began publishing artificial intelligence studies in the field of Oncology in 2015. It began to rise after 2018. In the early part of 2020, Scientigfic Reports was leading in publishing articles in this area, but after 2020, Frontiers in Oncology took the lead. Figure 15 shows that Frontiers in Oncology published these studies at significantly higher rates compared to other Journals.

### Web of science categories

According to the categorization within the Web of Science database, the articles were distributed across 147 scientific categories. However, nearly 70% of these articles predominantly fall within the scope of 10 major categories: Oncology (22%), Radiology, Nuclear Medicine Medical Imaging (13.4%), Engineering Biomedical (5.1%), Mathematical Computational Biology (5%), Engineering Electrical Electronic (4.8%), Computer Science Artificial Intelligence (4.7%), Computer Science Interdisciplinary Applications (4.7%) and Computer Science Information Systems (4.6%). Detailed category distribution is provided below (Table 4).

**Table 4:** Distribution of articles by top 20 web of science categories

Name	Web of Science Documents	Times Cited	Citation Impact	Documents in Q1 Journals	Documents in Q2 Journals	Documents in Q3 Journals	Documents in Q4 Journals
ONCOLOGY	2021	49,877	0.04	538	1006	195	114
RADIOLOGY, NUCLEAR MEDICINE & MEDICAL IMAGING	1774	53,085	0.03	763	550	239	51
ENGINEERING, BIOMEDICAL	493	14,545	0.03	227	151	59	21
COMPUTER SCIENCE, ARTIFICIAL INTELLIGENCE	490	19,532	0.03	159	136	58	33
MATHEMATICAL & COMPUTATIONAL BIOLOGY	477	8409	0.06	242	82	22	32
COMPUTER SCIENCE, INTERDISCIPLINARY APPLICATIONS	459	14,204	0.03	276	83	39	10
ENGINEERING, ELECTRICAL & ELECTRONIC	440	10,731	0.04	98	232	77	15
COMPUTER SCIENCE, INFORMATION SYSTEMS	418	6664	0.06	91	137	105	20
MEDICAL INFORMATICS	322	8318	0.04	147	67	59	11
BIOCHEMICAL RESEARCH METHODS	307	6115	0.05	125	111	40	18
BIOTECHNOLOGY & APPLIED MICROBIOLOGY	268	5080	0.05	100	59	61	7
MEDICINE, RESEARCH & EXPERIMENTAL	246	12,342	0.02	88	38	44	28
BIOCHEMISTRY & MOLECULAR BIOLOGY	241	12,785	0.02	119	75	31	9
COMPUTER SCIENCE, THEORY & METHODS	236	5488	0.04	65	90	20	3
HEALTH CARE SCIENCES & SERVICES	225	3944	0.06	76	75	31	11
GASTROENTEROLOGY & HEPATOLOGY	217	5324	0.04	84	60	31	21
CHEMISTRY, MULTIDISCIPLINARY	201	2710	0.07	27	82	86	2
SURGERY	193	2553	0.08	90	61	20	9
GENETICS & HEREDITY	165	2500	0.07	54	78	20	4
BIOLOGY	163	3630	0.04	101	38	16	3

InCites (2023)

## Keywords

**Fig. 9:**
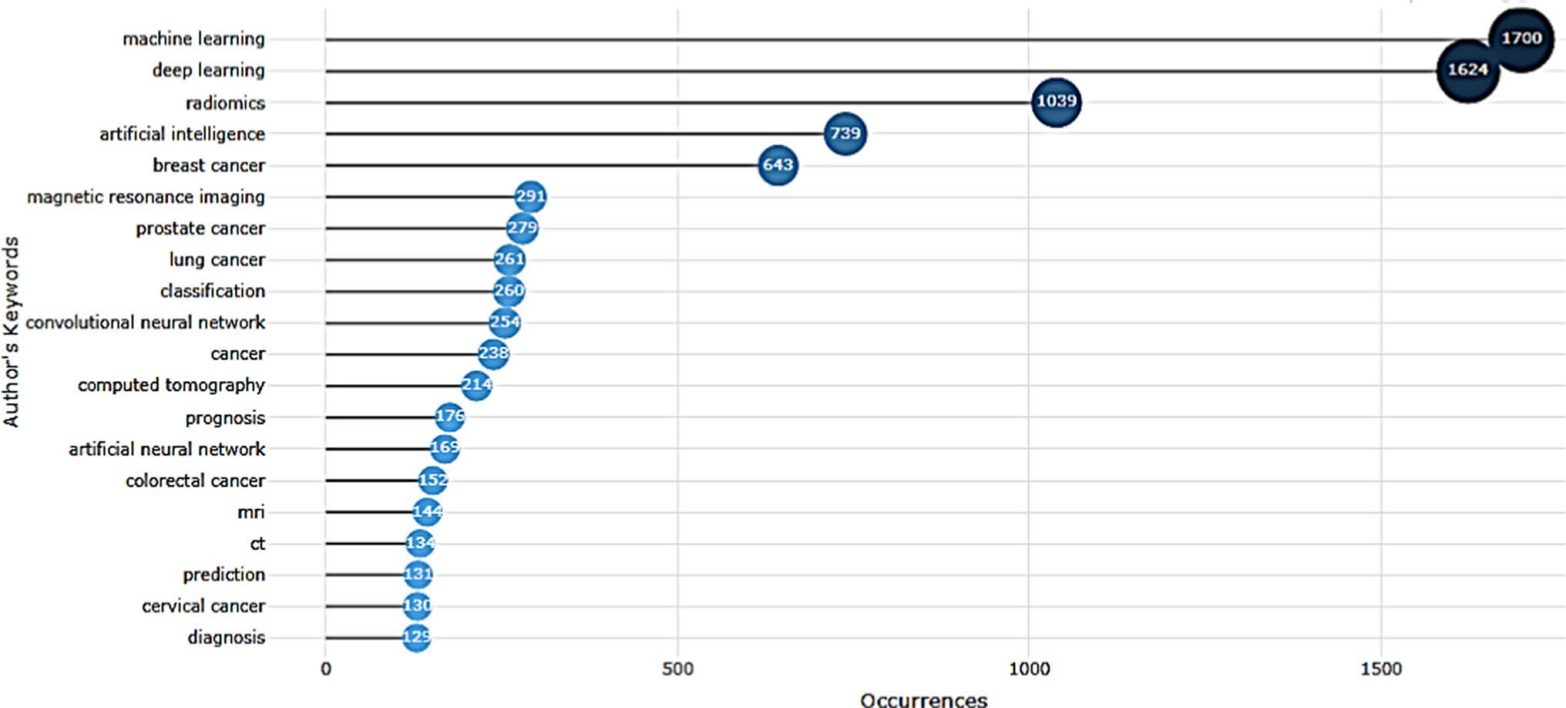
Most Frequent Author Keywords in the AI and Oncology literature, 1992–2022 (Bibliometrix & R software, 2023)

The most frequent author keyword analysis was conducted to identify research hotspots and future research directions in the academic field. In this study, authors’ keyword co-occurrence visualization graph was created by the Bibliometrix & R software program (Fig. 9). The top 20 author keywords were shown. The most frequent author keywords in the dataset are machine learning, deep learning, radiomics, artificial intelligence, breast cancer, magnetic resonance imaging, and prostate cancer (Fig. 9).

**Fig. 10:**
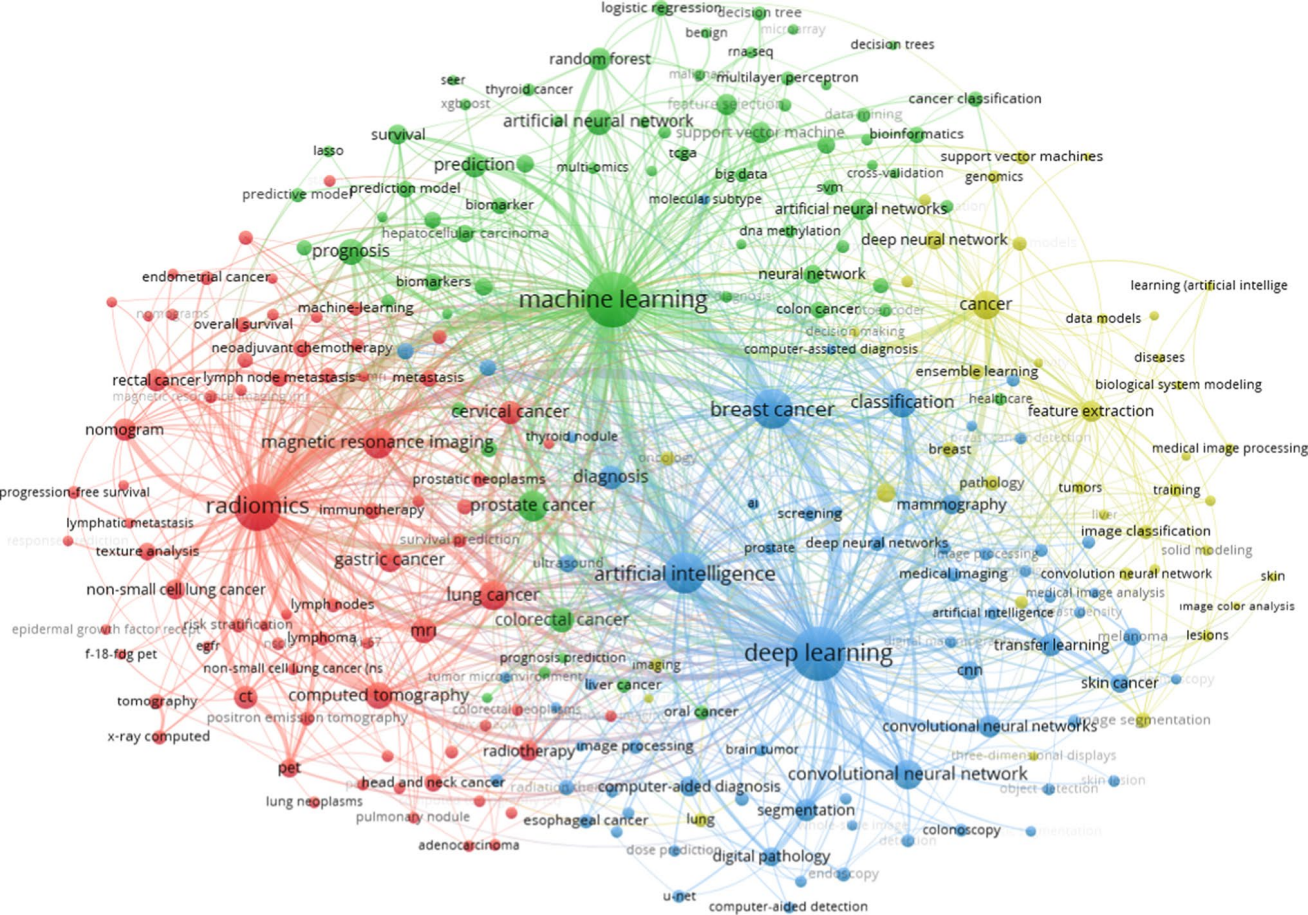
Network map of author keywords co-occurrence that appeared 12+ times in the artificial intelligence and Oncology literature, 1992–2022 (VOSviever, 2023)

Keyword co-occurrence analysis serves as a method to comprehend the primary themes or topics within a research field. Co-occurrence signifies the joint appearance of two pieces of information within a dataset. Each keyword within the dataset is represented as a node, while the co-occurrence of a pair of keywords is depicted as a link. The strength of this link is determined by the frequency of appearance of the paired keywords together [39]. This study employs Author Keywords for conducting keyword co-occurrence analysis. Author Keywords are automatically generated using a proprietary algorithm unique to Clarivate Analytics databases. The keywords associated with Artificial Intelligence (AI) applications in Oncology research are categorized into four clusters, denoted by four distinct colors (Fig. 10). Each circle within the figure represents a keyword, and the lines connecting these circles signify the connections between the keywords. Keywords sharing the same color belong to the same cluster. The size of each circle in the figure corresponds to the frequency of the keyword: larger circles indicate higher frequency, while smaller circles denote lower frequency.

Figure 10 shows the visualization network map of author keywords co-occurrence. Four Clusters are formed weights based on occurrences. The red color indicates Cluster 1 (radiomics, magnetic resonance imaging, computer tomograph, etc.); the green color indicates Cluster 2 (machine learning, prediction, artificial neural network, etc.); the blue color indicates Cluster 3 (deep learning, breast cancer, artificial intelligence, etc.); the yellow color indicates Cluster 4 (cancer, feature extraction, image classification, etc.).

### Countries and universities

**Fig. 11:**
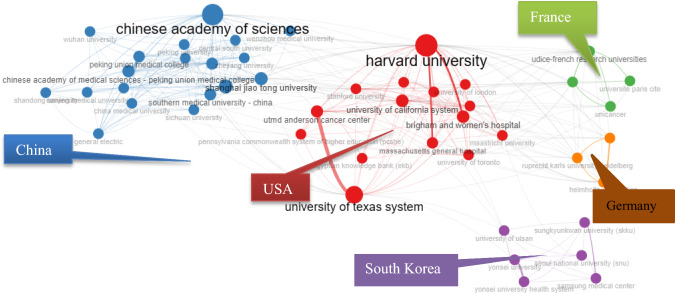
Network of institutions among articles with authors affiliated with institutions (Bibliometrix & R software, 2023)

Figure 11 shows the institution collaboration network based on authors. five main clusters of institutions were identified: mostly universities from China (see blue) and universities from the USA (see red). Chinese institutes constitute a majority of the entities involved, with prominent institutions like the Chinese Academy of Science and Shanghai Jiao Tong University standing out. These institutions are noted for their high activity and collaboration in the realm of AI articles related to oncology. The USA is the second-largest cluster developed in the collaboration network. The institutes in this cluster, including Harvard University and the University of Texas System, are the main ones.

**Fig. 12:**
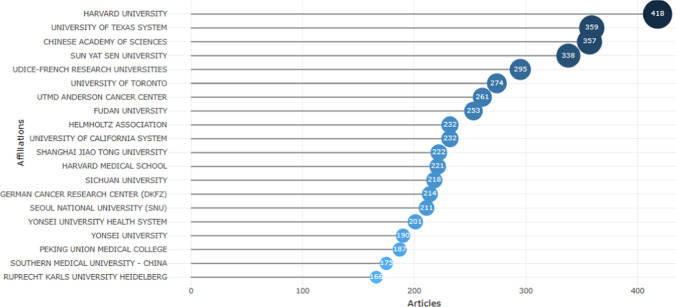
Distribution of author affiliations to organizations based on the selected dataset (top 20 organizations) (Bibliometrix & R software, 2023)

Figure 12 shows the top 20 institutions in terms of publications. The co-authorship analysis showed that 51 institutions published more than five papers.

**Fig. 13:**
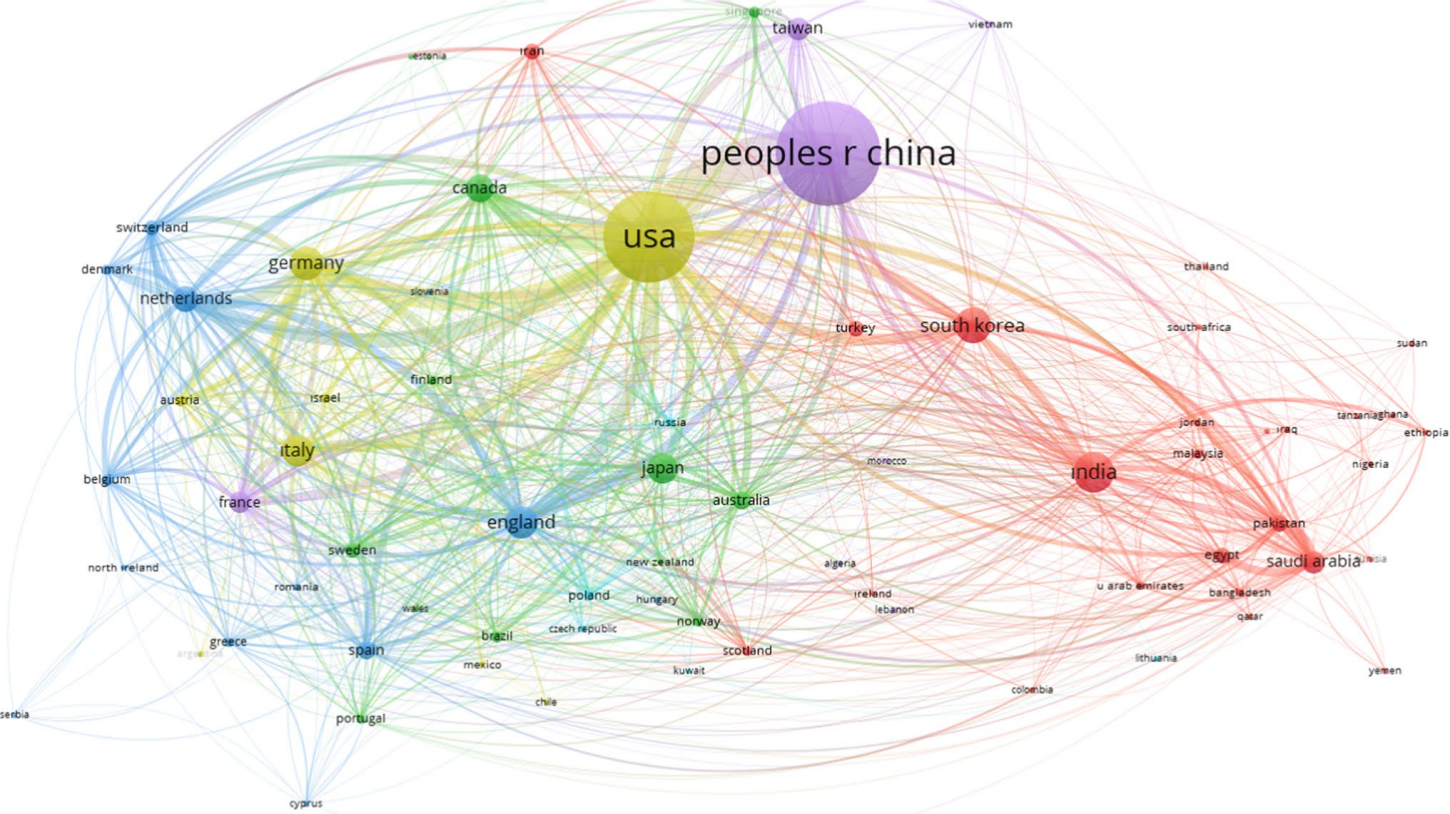
Co-authorship country visualization network map (Wosviever, 2023)

Figure 13 shows that only five clusters are formed; purple color indicates Cluster 1 (China, France, Taiwan, etc.); yellow color indicates Cluster 2 (USA, Germany, Italy, etc.); blue color indicates Cluster 3 (Netherlands, England, Spain, etc.); red color indicates Cluster 3 (South Korea, India, Egypt, Saudi Arabia, etc.); green color indicates cluster 5 (Japan, Australia, Norway, etc.)

**Fig. 14:**
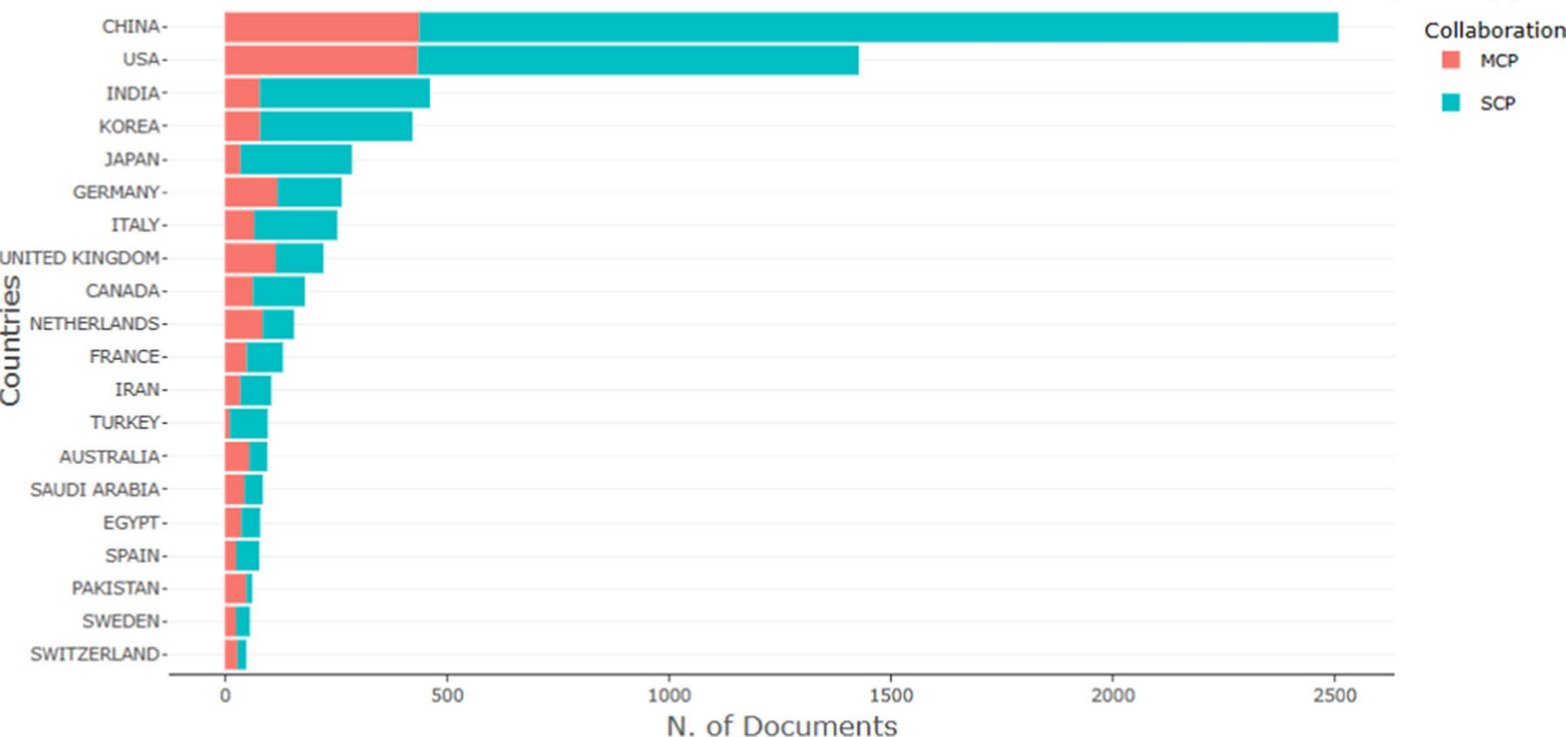
Most productive countries: Single Country Publications (SCP), Multiple Country Publications (MCP) (Bibliometrix & R software, 2023)

From the country/region perspective, 106 countries/regions have participated in the article publications. China involves 1001 articles, accounting for 28.34% of the total publications, followed by the USA (25.62%), India (6.63%), South Korea (6.40%), and the United Kingdom (5.63%) (Fig. 14).

Figure 14 presents the top 20 countries based on the number of articles published, with a categorization by Multiple Country Publication (MCP) and Single Country Publication (SCP). Multiple Country Publication refers to collaborative works involving authors from different countries, indicating international collaboration. Conversely, Single Country Publication denotes works where all authors belong to the same country, signifying intra-country collaboration. Chinese authors lead in productivity with 2508 articles, comprising 2071 single-country publications and 437 multiple-country publications, resulting in an MCP ratio of 17.4%. On the other hand, authors from Turkey exhibit the lowest ratio of multiple-country (collaboration with another country) publications, contributing a total of 96 articles, with only 11 being single-country publications (without collaboration another country). For more detailed information, refer to Fig. 14 and Table 5.

**Table 5:** Corresponding author's country and between-country collaboration

Country	Articles	SCP	MCP	Freq	MCP_Ratio
CHINA	2508	2071	437	0.323	0.174
USA	1428	994	434	0.184	0.304
INDIA	461	383	78	0.059	0.169
KOREA	422	343	79	0.054	0.187
JAPAN	286	251	35	0.037	0.122
GERMANY	263	145	118	0.034	0.449
ITALY	253	187	66	0.033	0.261
UNITED KINGDOM	222	108	114	0.029	0.514
CANADA	180	117	63	0.023	0.35
NETHERLANDS	155	70	85	0.02	0.548
FRANCE	130	81	49	0.017	0.377
IRAN	104	69	35	0.013	0.337
TURKEY	96	85	11	0.012	0.115
AUSTRALIA	95	40	55	0.012	0.579
SAUDI ARABIA	85	41	44	0.011	0.518
EGYPT	79	42	37	0.01	0.468
SPAIN	77	52	25	0.01	0.325
PAKISTAN	61	13	48	0.008	0.787
SWEDEN	56	31	25	0.007	0.446
SWITZERLAND	48	20	28	0.006	0.583

Bibliometrix & R software (2023)

Figure 14 further illustrates that the majority of publications are authored by individuals from the same countries. This trend might arise from authors' preferences to collaborate within their research groups or with academics sharing the same national background.

### Funding

According to the results presented in Table 6, the National Natural Science Foundation, with 1045 studies, and the Department of Health & Human Services, with 656 studies, had the highest support for the publication of scientific research on Artificial Intelligence for cancer detection. The National Natural Science Foundation, Department of Health & Human Services, NIH National Cancer Institute, US Department of Health and Human Services significantly improve artificial intelligence for cancer detection.

**Table 6:** Distribution of Articles according to the international organizations that funded them

Funding	Web of Science Documents	Times Cited	International Collaborations	Domestic Collaborations	Documents in Q1 Journals	Documents in Q2 Journals	Documents in Q3 Journals	Documents in Q4 Journals
National Natural Science Foundation of China	1045	18,886	229	626	472	417	109	22
Department of Health & Human Services-USA	656	22,221	251	264	353	196	34	6
National Institutes of Health (NIH)-USA	649	21,919	248	263	350	195	33	6
NIH National Cancer Institute (NCI)-USA	270	9855	97	118	141	81	15	2
National Research Foundation of Korea	205	2843	35	130	96	83	8	12
National Science Foundation-USA	102	2549	43	35	56	24	6	1
Ministry of Education Culture Sports Science and Technology-Japan	89	1155	29	45	31	39	9	2
Japan Society for the Promotion of Science	87	1121	29	43	31	38	9	2
Ministry of Science ICT & Future Planning-Republic of Korea	86	1155	15	56	40	37	3	3
European Union-EU	83	1964	56	18	43	29	3	1
Fundamental Research Funds for the Central Universities-China	81	1789	25	41	36	34	10	0
National Natural Science Foundation of Guangdong Province-China	77	1665	11	56	36	30	6	2
Grants-in-Aid for Scientific Research-Japan	72	895	20	38	24	33	8	1
UK Research & Innovation-UK	71	3290	41	24	45	18	0	1
Beijing Natural Science Foundation-China	68	1818	16	46	32	31	5	0
Spanish Government	66	3096	29	25	42	18	2	0
Ministry of Science and Technology-Taiwan	64	960	12	49	34	23	6	0
German Research Foundation (DFG)-Germany	61	2074	35	23	40	15	3	1
Ministry of Science & ICT-Republic of Korea	61	733	11	35	26	27	4	1
China Postdoctoral Science Foundation	60	674	16	38	34	24	1	0
Natural Sciences and Engineering Research Council of Canada	56	1602	20	25	26	21	4	0
European Research Council	45	3609	31	8	30	13	0	0
Medical Research Council UK	44	2902	25	17	29	11	0	1
Natural Science Foundation of Zhejiang Province-China	42	713	18	21	20	19	3	0
Canadian Institutes of Health Research	40	1379	11	24	21	13	0	0

InCites (2023)

**Fig. 15:**
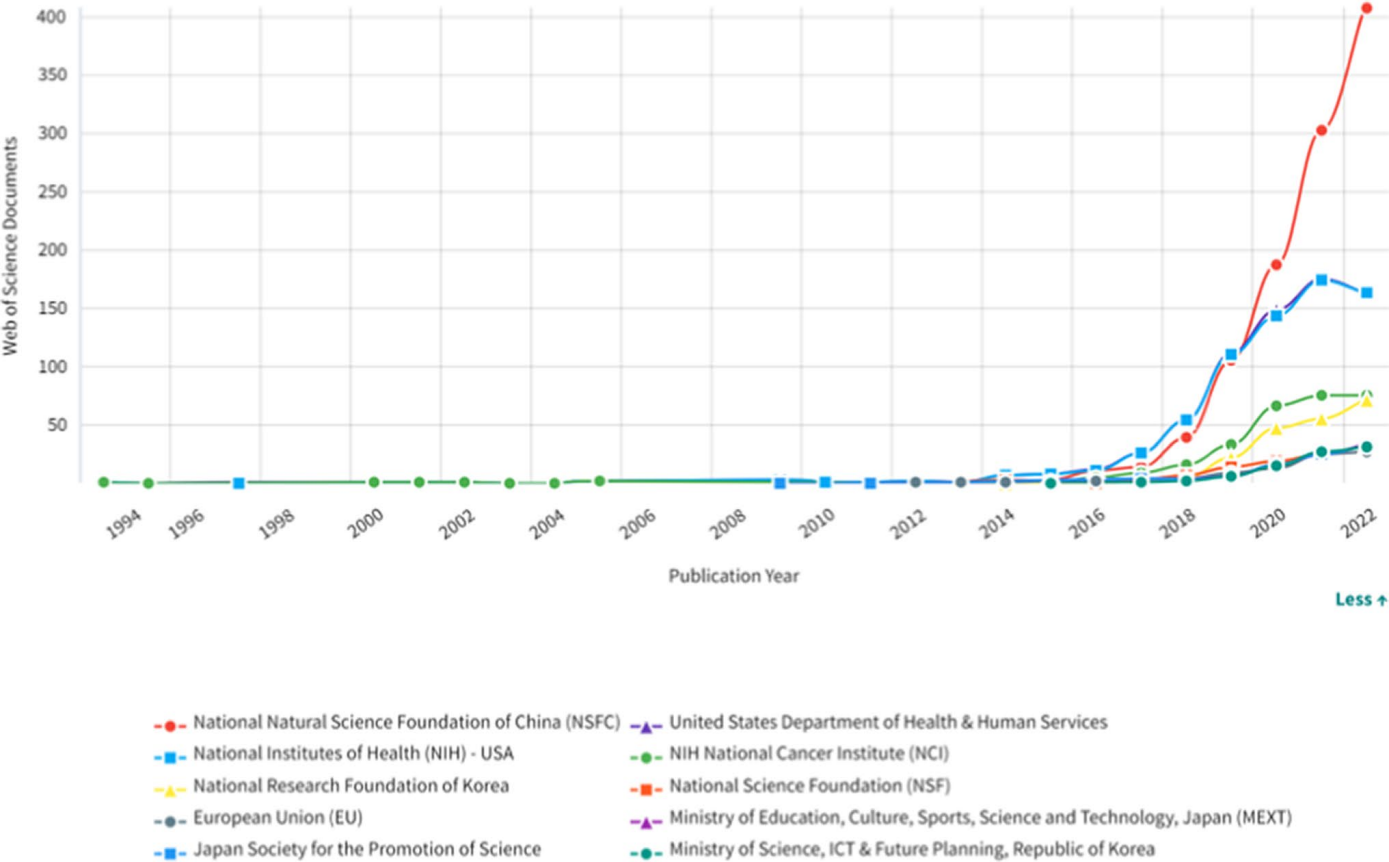
Changes in the distribution of the articles according to the institutions supporting the articles by years (InCites, 2023)

As shown in Fig. 15, countries began supporting artificial intelligence studies in the field of Oncology in 2014. It began to rise after 2016. In the early part of 2016, the National Institutes of Health (NIH) in the USA was leading in supporting studies in this area, but after 2019, the National Natural Science Foundation of China (NSFC) took the lead. Figure 15 shows that NSFC supported these studies at significantly higher rates compared to other funding organizations. It's noticeable that the expected support from NIH's National Cancer Institute lagged behind. As seen in previous graphs and tables, it demonstrates how extensively China has invested in this field.

## Discussion

In recent years, artificial intelligence (AI) has swiftly become an integral part of the medical field, particularly in cancer detection. This study utilized the bibliometrix package of R software and Litmaps visualization software to conduct a thorough bibliometric analysis of AI applications in oncology research over the past 30 years. The objective was to provide a comprehensive understanding of the field. Our analysis objectively and systematically outlined the current status of AI applications, identified developmental trends, and highlighted potential research focal points in cancer detection. This endeavor facilitates scholars in rapidly comprehending the research landscape and offers valuable insights for selecting research topics. The initial phase of the study examined publication trends, covering aspects such as countries, institutions, authors, and journals. Subsequently, cluster analysis was applied to keywords to identify research hotspots within the field.

Based on the analysis of publication trends, there has been a significant surge in the number of publications on AI in Oncology over the past four years. China and the United States emerged as the leading nations regarding the volume of publications in this field. Citation counts, widely recognized as an indicator of professional acknowledgment in scientific work, were extensively used to assess research quality. The United States stood out in terms of both citation counts and international collaborations, with a considerable lead over other countries. Additionally, the university contributing the most publications and citations was based in China, underscoring China's pivotal role and global leadership in this domain. Despite China's considerable volume of publications, the relatively low citation counts suggest a need to enhance the quality and impact of its research [40]. This could be attributed, in part, to the later initiation of AI in Oncology research in China, resulting in comparatively lower international academic influence. Notably, Jie Tian and Zaiyi Liu from China emerged as the most published authors, contributing to 10.3% of the publications. Regarding citations, Hugo Aerts from Stanford University and Robyn Gillies from the University of Washington in the United States received the highest recognition. These findings underscore the importance of quantity, quality, and global impact in advancing AI research in cancer detection.

This is the newest bibliometric study that provides detailed information about published literature on the AI in Oncology. The most active institutions were the Chinese Academy of Science and Harward University, and the most productive countries were China and the USA. The most frequently co-occurrence author keywords were radiomics, machine learning, artificial intelligence, and breast cancer.

This bibliometric study holds potential by offering a comprehensive overview of AI in oncology, identifying research hotspots like radiomics and deep learning, and highlighting future research directions such as extracting meaning from genomic/proteomic/clinicomic data. Significant knowledge gaps exist in effectively utilizing these diverse data types and integrating AI seamlessly into clinical workflows, alongside addressing ethical considerations. Researchers are tackling these challenges through advanced algorithms, improved data standardization, and collaborative efforts to develop user-friendly tools. Over the next five years, the field will likely see increased focus on clinicomics and multi-omics, advancements in early cancer detection and prevention, personalized treatment planning, AI-powered drug discovery, and wider adoption of AI tools in clinical settings, fostered by greater collaboration and standardization across the field.

The outcomes of this study hold value for researchers, policymakers, and educational purposes. Additionally, they offer assistance to funding agencies in evaluating current research trajectories and anticipating future trends in AI within Oncology. Effective AI development and treatment therapy is still a hot zone for future research directions. Three leading journals publish the most articles on artificial intelligence and Oncology: Frontiers in Oncology (428 articles) and Scientific Reports and Cancer (284 and 247 articles, respectively). While these three leading journals accounted for 12% of total articles, the remaining list is well-distributed. The majority, almost 70%, of the articles fit into ten significant categories: Oncology (22%), Radiology Nuclear Medicine Medical Imaging (13.4%), Engineering Biomedical (5.1%), Mathematical Computational Biology (5%),

## Limitations

This study has some limitations that should be acknowledged. One of the main limitations is the reliance on WoS and InCites as the sole data sources. While these databases are widely wellknown as authoritative sources of citation data, they may not capture the full range of publications and citations in the field. The exclusion of other data sources, such as Scopus, Pubmed, Google Scholar, or non-English language publications, may have introduced biases into our analysis.

## Conclusion

Articles about extracting meaning from radiological/microscopic/real patient images in cancer patients using artificial intelligence have been produced for approximately 6 years and have reached a certain maturity. It is understood that some obstacles are related to deriving meaning from genomic/genomic/proteomic data and doctor notes written in text. As new findings emerge, the association of diseases and treatments using existing classification systems with genomic/proteomic data should be expected to increase geometrically/exponentially. In order to derive meaning from clinical data, it is necessary to create new databases of the NoSQL type for transferring values from biochemistry, tumor markers, drug doses, as well as names of drugs/materials/devices, and text notes written manually with pen or keyboard into artificial intelligence software. Therefore, it is expected that big data derived from electronic health records should be reprocessed and re-archived by developing new standards. It is understood that the CancerLinq database has not contributed to machine learning-related publications so far and may not be able to do so in its current state. It is observed that despite fewer available parameters for individuals in the previously established SEER database, more publications related to machine learning have been made. This situation may stem from a structural difference between the CancerLinq and SEER databases.

The exploration of artificial intelligence in Oncology is in its early phases but is anticipated to progress rapidly. Researchers are currently investigating AI applications in various aspects of Oncology within the medical field, including medicine, diagnosis, therapy, and risk assessment. Implementing artificial intelligence proves effective in mitigating human errors and enhancing work efficiency. This bibliometric study offers a comprehensive overview of AI in Oncology research, focusing on the discipline's current state. This perspective assists researchers in identifying critical areas of interest, cutting-edge developments, and emerging research directions within the field.

## Data Availability

The datasets used and/or analysed during the current study available from the corresponding author on reasonable request.
